# A novel water‐in‐oil emulsion with a lecithin‐modified bentonite prevents skin damage from urban dust and cedar pollen

**DOI:** 10.1111/ics.12605

**Published:** 2020-04-26

**Authors:** T. Iwanaga, A. Nioh, N. Reed, H. Kiyokawa, H. Akatsuka

**Affiliations:** ^1^ Frontier Research Center Pola Chemical Industries, Inc. Kashio‐cho 560, Totsuka‐ku Yokohama Japan; ^2^ Jurlique International Pty. Ltd. 44‐50 Oborn Road Mount Barker South Australia Australia

**Keywords:** emulsions, oxidative stress, skin ageing, skin barrier, skin physiology, structure

## Abstract

**Objective:**

Particulate matter (PM), such as air pollutants and pollens, are known to cause skin ageing through skin inflammation. It is important to develop formulations which protect the skin from PM. We previously developed a conventional water‐in‐oil emulsion with a synthetic surfactant, distearyldimonium chloride, modified bentonite (C‐W/O), which protects skin from allergens. In the present study, we developed a novel water‐in‐oil emulsion with a natural surfactant, lecithin, modified bentonite (N‐W/O).

**Methods:**

The microarray analysis was performed using total RNA extracted from a reconstructed human epidermis (RHE) stimulated with urban aerosols or cedar pollen for 6 h in order to develop an epidermal inflammation model by PM for the evaluation of topical formulations. We then compared the efficacy of N‐W/O and C‐W/O to prevent epidermal degradation. Tissues and culture media were collected 24 h after the urban aerosol or cedar pollen stimulation for a histological assay, and the quantification of MMP1 and IL‐8 secretion.

**Results:**

The expression levels of proinflammatory cytokines and chemokines, such as *IL1A* and *CXCL8*, and matrix metalloproteinases, including *MMP1*, *MMP3* and *MMP9*, were significantly up‐regulated by the PM stimulation. As a result of ranking based on the pathway enrichment analysis, oxidative stress‐related pathways, such as MAPK‐mediated signalling, HIF‐1 signalling, IL‐1 signalling and ROS‐induced cellular signalling, were ranked high in the urban dust‐ and cedar pollen‐treated groups. A thickened stratum corneum, thinned vital layer and cleaved E‐cadherin were observed by haematoxylin and eosin staining and immunohistochemical staining of E‐cadherin in the PM treated groups. The secretion of MMP1 and IL‐8 into the media was significantly increased by the PM stimulation. N‐W/O prevented the degradation of epidermal integrity and secretion of inflammatory proteins more effectively than C‐W/O.

**Conclusion:**

The present results showed that N‐W/O made using natural surfactant is useful at protecting skin from PM, such as urban aerosols and cedar pollen.

## Graphical abstract

A thickened stratum corneum, thinned vital layer and cleaved E‐cadherin were observed by the exposure of urban dust and cedar pollen to reconstructed human epidermis. A novel water‐in‐oil emulsion with a natural surfactant, lecithin, modified bentonite (N‐W/O) significantly prevented these inflammation. N‐W/O made using natural surfactant is useful at protecting skin from particulate matter, such as urban aerosols and cedar pollen.

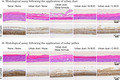

## Résumé

**Objectif:**

Les particules en suspensions (PM), telles que les polluants atmosphériques et les pollens, sont connues comme des causes de vieillissement de la peau par inflammation cutanée. Il est essentiel de mettre au point des formules qui protègent la peau contre ces particules. Par le passé, nous avons mis au point une émulsion eau‐dans‐huile classique composée d’un tensioactif synthétique, de distearyldimonium chloride et de bentonites modifiées (E/H‐C), qui protège la peau contre les allergènes. Dans la présente étude, nous avons conçu une nouvelle émulsion eau‐dans‐huile composée d’un tensioactif naturel, de lécithine et de bentonites modifiées (N‐E/H).

**Méthodes:**

L’analyse des microréseaux a été réalisée à l’aide de l’ARN total extrait d’un épiderme humain reconstitué (EHR) stimulé par les aérosols urbains ou le pollen de cèdre pendant 6 h afin de mettre au point un modèle d’inflammation de l’épiderme par les particules en suspensions en vue de l’évaluation des formulations topiques. Nous avons ensuite comparé l’efficacité de la N‐E/H et de l’E/H‐C dans le but d’éviter la dégradation de la peau. Les milieux de culture tissulaire ont été collectés 24 h après stimulation par l’aérosol urbain ou par du pollen de cèdre pour un dosage histologique et une quantification de MMP‐1 et des sécrétions de l’IL‐8.

**Résultats:**

Les niveaux d’expression des cytokines pro‐inflammatoires et des chimiokines, à l’instar de l’IL1A et du CXCL8, ainsi que des métalloprotéinases matricielles, notamment les MMP1, les MMP3 et les MMP9, étaient essentiellement régulés positivement par la stimulation des particules en suspensions. En raison du classement basé sur l’analyse d’enrichissement des voies, le stress oxydatif, telles que la signalisation médiée par MAPK, la signalisation HIF‐1, la signalisation IL‐1 et la signalisation cellulaire induite par les ROS ont été classés en tête pour les groupes traités par la poussière urbaine et par le pollen‐de cèdre. Un stratum corneum épaissie, une couche vitale fine et une clivée d’E‐cadhérine ont été observées par coloration à l’hématoxyline‐éosine et par coloration immunohistochimique de l’E‐cadhérine dans les groupes traités aux particules en suspensions. La sécrétion de MMP1 et de l’IL‐8 dans les milieux a augmenté de façon significative par stimulation des particules en suspensions. La N‐E/H a permis d’éviter une dégradation de l’intégrité de la peau et la sécrétion de protéines inflammatoires de manière plus efficace que l’E/H‐C.

**Conclusion:**

Les résultats actuels ont révélé que la N‐E/H produite grâce à l’utilisation d’un tensioactif naturel est utile pour la protection de la peau contre les particules en suspensions telles que les aérosols urbains et le pollen de cèdre.

## Introduction

Particulate matter (PM) is a widespread air pollutant that comprises a mixture of particulate contaminants (including smog, tobacco smoke and soot), various types of dust, biological contaminants (such as pollen and house dust mite allergens) and gaseous contaminants (including exhaust gas from traffic). PM exerts adverse effects on human health by, for example, increasing the risk of cancer and pulmonary and cardiovascular diseases 1, 2, 3. Recent studies reported that PM has a negative impact on skin and also induces extrinsic skin ageing, including wrinkling and pigmentation 4, 5, 6. One of mechanisms by which PM induces skin ageing is by triggering the formation of intracellular reactive oxygen species (ROS) through mitochondrial damage and redox cycling and directly through particle surface reactivity 7. Increases in ROS concentrations inhibit collagen synthesis as a result of the activation of matrix metalloproteases (MMPs) 8, 9. Furthermore, PM has been shown to enhance the production of proinflammatory cytokines and chemokines, such as IL‐1α and IL‐8, by human keratinocytes 10, 11. Since these PM‐induced reactions lead to skin ageing, it is important to develop the formulations for the protection of skin from exposure to PM. These formulations should be made from natural ingredients including biosurfactants because they have low toxicity, biodegradability and higher stability under a wide range of physicochemical environments than synthetic surfactants 12.

In the present study, we used a reconstructed human epidermis (RHE) with PM‐induced inflammation (urban aerosol and cedar pollen) to evaluate the protection efficacy of a novel water‐in‐oil emulsion with a natural surfactant, lecithin, modified bentonite (N‐W/O). We compared N‐W/O with a conventional water‐in‐oil emulsion with a synthetic surfactant, distearyldimonium chloride, modified bentonite (C‐W/O), which was previously demonstrated to protect skin from allergens more effectively than petrolatum 13.

## Materials and methods

### RHE model

A three‐dimensional RHE model, LabCyte EPI‐MODEL (Japan Tissue Engineering Co., Ltd., Aichi, Japan), which is composed of normal human epidermal keratinocytes that form a multilayered structure, was maintained according to the manufacturer’s instructions.

### Particulate matter

Urban dust collected on filters from the central ventilating system of a building in the centre of Beijing city between 1996 and 2005 (CRM No. 28, National Institute for Environmental Studies, Ibaraki, Japan) and Japanese cedar pollen (FUJIFILM Wako Pure Chemical Corporation, Osaka, Japan) were used in the present study.

### Microarray analysis of the RHE inflammation model with PM exposure

RHE was exposed to 25 mg of urban dust or cedar pollen, while control samples were not. After six hours of PM exposure, total RNA was extracted from RHE using TRIzol® reagent (Thermo Fisher Scientific, MA, USA) and the RNeasy Mini Kit (Qiagen, Tokyo, Japan) modified from methods described in a previous study 14. Briefly, all samples were homogenized using Biomasher® II (Nippi Inc., Tokyo, Japan) with 700 μL TRIzol® reagent, and 140 μL chloroform was added. A total of 350 μL of the aqueous layer (containing RNA) was transferred to new RNase‐free tubes after centrifugation at 12 000 *g* at 4 °C for 15 min. The aqueous layer was added to the same volume of 70% ethanol, and immediately mixed by pipetting. The mixture was transferred to an RNeasy spin column placed in a 2‐mL collection tube and subjected to total RNA extraction according to the manufacturer’s instructions. The quality and concentration of total RNA were assessed using a Nanodrop ND‐1000 spectrometer (Thermo Fisher Scientific, MA, USA). Total RNA obtained was used in a DNA microarray analysis with SurePrint G3 8x60K Microarrays (Agilent Technologies, Inc., CA, USA) as described previously 15. The Agilent protocol ‘One‐Color Microarray‐Based Gene Expression Analysis (Low Input Quick Amp Labeling), Ver6.9, December 2015’ was used for sample preparation and array processing. Cy3‐labelled cRNA was subjected to hybridization by an incubation in a hybridization oven (Agilent Technologies, Inc.) for 17 h. Hybridized slides were scanned with the G2505C scanner (Agilent Technologies, Inc.), and data were obtained using Agilent Feature Extraction software (version 10.7.1.1, Agilent Technologies, Inc.) with defaults for all parameters. Microarray data analyses were performed using GeneSpring GX (version 14.5) software (Agilent Technologies, Inc.).

The significance of differences in gene expression between the control and treated groups was assessed using Welch’s *t*‐test. Differentially expressed genes (DEGs) were defined according to the following three criteria: (i) Significance level, *P* < 0.05. The gene expression ratio is >1.5 or <0.67, and all flags of each sample are ‘Detected’. (ii) Significance level, *P* < 0.01. The gene expression ratio is 1–1.5 or 0.67–1, and the flag of each sample is ‘Detected’. (iiia) Significance level, *P* < 0.01. The gene expression ratio is >4, including the ‘Not Detected’ flag in the control group. (iiib) Significance level, *P* < 0.01. The gene expression ratio is <0.25, including the ‘Not Detected’ flag in the exposure group. DEGs were imported for a pathway enrichment analysis into MetaCore® software (Thomson Reuters, NY, USA).

### Materials including emulsion samples (Table 1)

N‐W/O was formulated using a lecithin‐modified bentonite made from hydrogenated lecithins (Lucas Meyer Cosmetics Inc., Champlan, France) and bentonites (HOJUN Co., Ltd., Gunma, Japan). The synthetic surfactant used to formulate C‐W/O was distearyldimonium‐modified bentonite (Bentone 38V, Elementis Specialties Inc., London, UK). Sorbitan fatty acid esters (Toho Chemical Industry Co., Ltd., Tokyo, Japan) and dimethicone copolyol (Shin‐Etsu Chemical Co., Ltd, Japan) were used as surfactants, and hydrocarbon (Amyris, Inc., California, USA) was used as the oil. Glycerine (Emery Oleochemicals Sdn Bhd., Selangor, Malaysia), a butylene glycol (Kokyu Alcohol Kogyo Co., LTD., Chiba, Japan), and ion‐exchanged water were used as aqueous bases. Commercial products other than lecithin bentonite were used without washing or purification.

**Table 1:** Formulation of emulsions

	Ingredients (w/w%)	Emulsion sample		
		Base formula	N‐W/O*	C‐W/O†
Modified clay	Lecithin‐modified bentonite		2.0	
	Distearyldimonium‐modified bentonite			2.0
Surfactant	Sorbitan fatty acid esters	2.5	0.5	
	Dimethicone copolyol			4.0
Oil	Hydrocarbon	8.0	32.0	32.0
Water phase	Glycerine	15.0	15.0	15.0
	Butylene glycol	6.0	6.0	6.0
	Water	68.5	44.5	41.0
Total		100.0	100.0	100.0

* Novel water‐in‐oil emulsion with a lecithin‐modified bentonite.

^†^ Conventional water‐in‐oil emulsion with a synthetic surfactant‐modified bentonite.

### Preparation of lecithin‐modified bentonite

A 1.75 (wt) % aqueous solution of hydrogenated lecithins was prepared, and citric acid (Iwata Chemical CO., LTD., Shizuoka, Japan) was added to pH 2.5–3.0. After pH‐adjusted hydrogenated lecithin solution had been temperature‐adjusted to 65 °C, 0.625 mol of bentonite was gradually added to 1 mol of hydrogenated lecithin. The mixture was stirred with a magnetic stirrer for 2 h. After stirring and cooling to room temperature, reactants were filtered by suction filtration and then washed once with an aqueous citric acid solution of pH 2.5 and three times with water to obtain a paste‐like filtrate. The washed filtrate was dried under decompression conditions (−1 Pa, 80 °C) for one day and then pulverized using a mixer to obtain a lecithin‐modified bentonite powder.

### Preparation of emulsion samples

The modified clay, surfactant and oil listed in Table 1 were mixed in a 100‐mL test tube and homogenized by stirring for 5 min at 2000 rpm in a homogenizer (NS‐360D, NS‐7, MICROTEC CO., LTD., Chiba, Japan). Pre‐mixed aqueous phase components were added and stirred with a homogenizer at 6000 rpm for 10 min to prepare creamy W/O emulsion samples. The base formula was prepared by hand stirring with a glass rod, and the internal water phase ratio was adjusted to achieve a similar creamy state to that of N‐W/O and C‐W/O.

### Evaluation of the efficacy of N‐W/O to protect against PM

RHE was treated with 100 μL of the base formula, N‐W/O or C‐W/O, before being exposed to 25 mg urban dust or cedar pollen, while control RHE was not exposed. Twenty‐four hours after the exposure to PM, culture media were collected to quantify protein content based on ELISA as follows: quantification of MMP1 (ab100603, Abcam Inc., MA, USA) and IL‐8 (D8000C, R&D Biosystems, MN, USA). MMP1 and IL‐8 in the media were quantified according to the manufacturer’s protocol. A statistical analysis was performed with a one‐way analysis of variance followed by the post‐hoc Tukey–Kramer test.

RHE was harvested after 24 h of PM exposure for the MTT assay (403026, Japan Tissue Engineering Co., Ltd., Aichi, Japan) or histological assays. Formaldehyde‐fixed RHE samples were embedded in paraffin blocks and stained with haematoxylin and eosin (H&E). And E‐cadherin was stained immunohistochemically using Novocastra™ Liquid Mouse Monoclonal Antibody E‐Cadherin (NCL‐L‐E‐Cad, Leica Biosystems, Wetzlar, Germany). The primary antibody was detected using the biotinylated secondary antibody, horseradish peroxidase‐labelled streptavidin and 3, 3′‐diaminobenzidine (ab64259, Abcam Inc.).

## Results

### Microarray analysis of the PM‐induced inflammation in RHE

**Figure 1:**
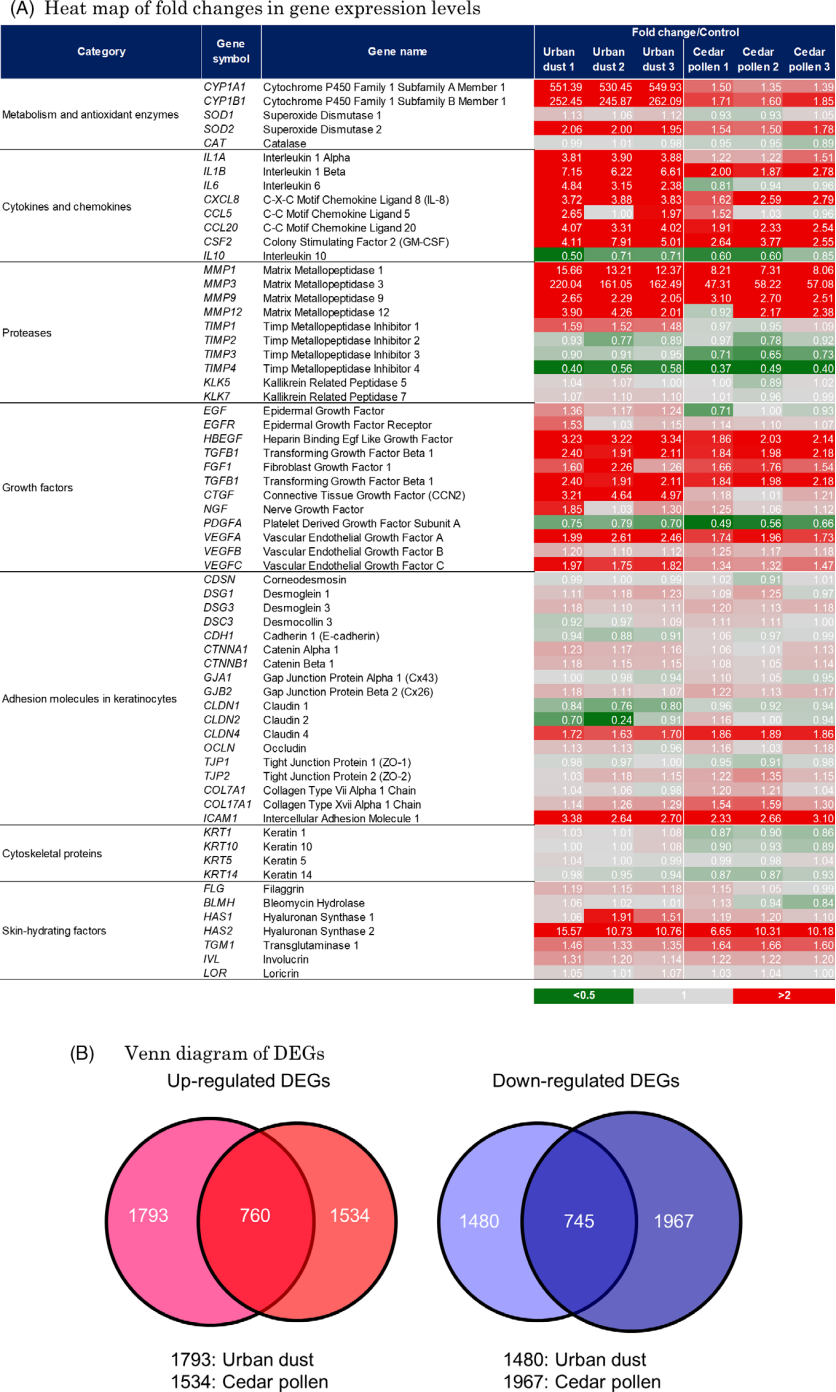
Microarray analysis of the reconstructed human epidermis model after 6 h of urban dust or cedar pollen exposure. (A) A heat map shows fold changes in gene expression levels in the urban dust‐ and cedar pollen‐treated groups from those in the control group. Many genes categorized as metabolism and antioxidant enzymes, cytokines and chemokines, proteases and growth factors were commonly up‐regulated following the exposure to urban dust and cedar pollen. The experiments were performed in triplicate, and each data was shown in fold change/control column. (B) Comparison of the number of differentially expressed genes (DEGs) between the urban dust‐ and cedar pollen‐treated groups was shown using a Venn diagram. Approximately 50% of up‐ or down‐regulated DEGs were similar in the urban dust‐ and cedar pollen‐treated groups.

Fold changes in gene expression levels in PM‐exposed samples from those in the control were shown using a heat map (Fig. 1a). Most genes categorized as metabolism and antioxidant enzymes, cytokines and chemokines, proteases and growth factors were commonly up‐regulated following the exposure to urban dust and cedar pollen. On the other hand, most genes categorized as adhesion molecules in keratinocytes, cytoskeletal proteins and skin‐hydrating factors maintained the same gene expression levels. The up‐regulated expression levels of metabolism and antioxidant enzymes, cytokines and chemokines, proteases and growth factors were higher in the urban dust‐treated group than in the cedar pollen‐treated group. Changes in *CYP1A1* and *CYP1B1* mRNA levels were 544‐ and 253‐fold higher, respectively, in the urban dust‐treated group than in the control group. These up‐regulated levels were markedly higher than those in the cedar pollen‐treated group. The numbers of up‐regulated DEGs in the urban dust‐ and cedar pollen‐treated groups were 1793 and 1534, respectively, whereas those of down‐regulated DEGs were 1480 and 1967, respectively. Approximately 50% of up‐ or down‐regulated DEGs were the same in the urban dust‐ and cedar pollen‐treated groups (Fig. 1b). As a result of ranking based on the pathway enrichment analysis by MetaCore® software, oxidative stress‐related pathways, such as MAPK‐mediated signalling, HIF‐1 signalling, IL‐1 signalling and ROS‐induced cellular signalling, were ranked high in the urban dust‐ and cedar pollen‐treated groups (Table 2).

**Table 2:** Ranking based on a pathway enrichment analysis using MetaCore® software

#	Maps	Total	Urban dust		Cedar pollen	
			*P*‐value	In Data	*P*‐value	In Data
1	Neurogenesis_NGF/TrkA MAPK‐mediated signalling	105	1.3E‐14	37	1.3E‐06	25
2	Transcription_HIF‐1 targets	95	9.7E‐11	30	6.8E‐14	34
3	Immune response_IL‐1 signalling pathway	82	2.0E‐13	31	3.2E‐08	24
4	Oxidative stress_ROS‐induced cellular signalling	108	3.0E‐11	33	1.1E‐08	29
5	Immune response_Lysophosphatidic acid signalling via NF‐kB	53	7.4E‐11	22	2.3E‐08	19
6	Chemotaxis_Lysophosphatidic acid signalling via GPCRs	129	3.0E‐10	35	1.0E‐03	22
7	TGF‐beta signalling via SMADs in breast cancer	47	3.2E‐10	20	1.1E‐07	17
8	Proinflammatory action of Gastrin in gastric cancer	50	1.2E‐09	20	1.8E‐04	13
9	FAK1 signalling in melanoma	42	2.1E‐09	18	2.0E‐03	10
10	IL‐1 signalling in melanoma	42	2.1E‐09	18	4.4E‐06	14

### Evaluation of protection by N‐W/O using the RHE inflammation model

**Figure 2:**
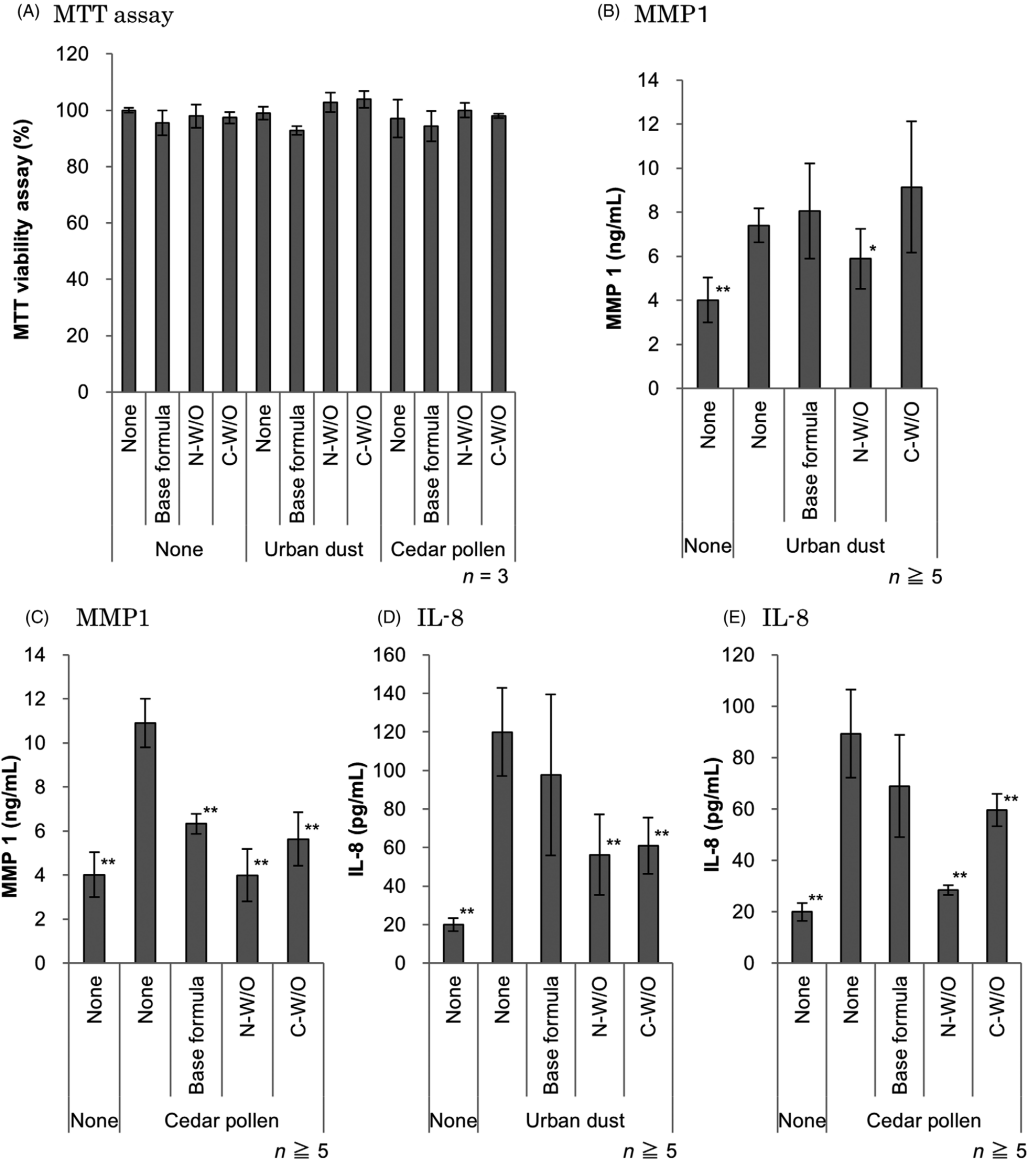
Evaluation of viability and preventative effects on MMP1 and IL‐8 secretion by a novel water‐in‐oil emulsion with a lecithin‐modified bentonite (N‐W/O) and a conventional water‐in‐oil emulsion with a synthetic surfactant‐modified bentonite (C‐W/O) after 24‐h exposure to urban dust or cedar pollen. (A) Viability was measured using the MTT assay. All samples maintained viability. (B‐E) Culture media were collected to quantify (B, C) MMP1 and (D, E) IL‐8. MMP1 and IL‐8 secretion was significantly induced by the application of urban dust or cedar pollen, and significantly prevented by the N‐W/O topical treatment. Data were presented as means ± SD. A statistical analysis was performed with a one‐way analysis of variance followed by the post‐hoc Tukey–Kramer test. The asterisks indicate statistical significance compared with the urban dust‐ or cedar pollen‐treated groups. *P < 0.05, **P < 0.01.

The viability of RHE samples after 24 h exposure to urban dust or cedar pollen with the application of no treatment, the base formula, N‐W/O and C‐W/O was measured using the MTT assay. None of the treated groups showed significant decreases in viability from the control group (Fig. 2a). MMP1 and IL‐8 secretion in the culture media significantly increased after 24‐h exposure to urban dust and cedar pollen. N‐W/O significantly prevented the up‐regulation of MMP1 and IL‐8 secretion compared with PM‐treated groups. In addition, the PM‐induced MMP1 and IL‐8 secretion amounts in the N‐W/O treated groups tended to be lower than C‐W/O treated groups, and the secretion amount of IL‐8 induced by cedar pollen in the N‐W/O treated groups was significantly lower than C‐W/O treated groups (Fig. 2b‐e).

**Figure 3:**
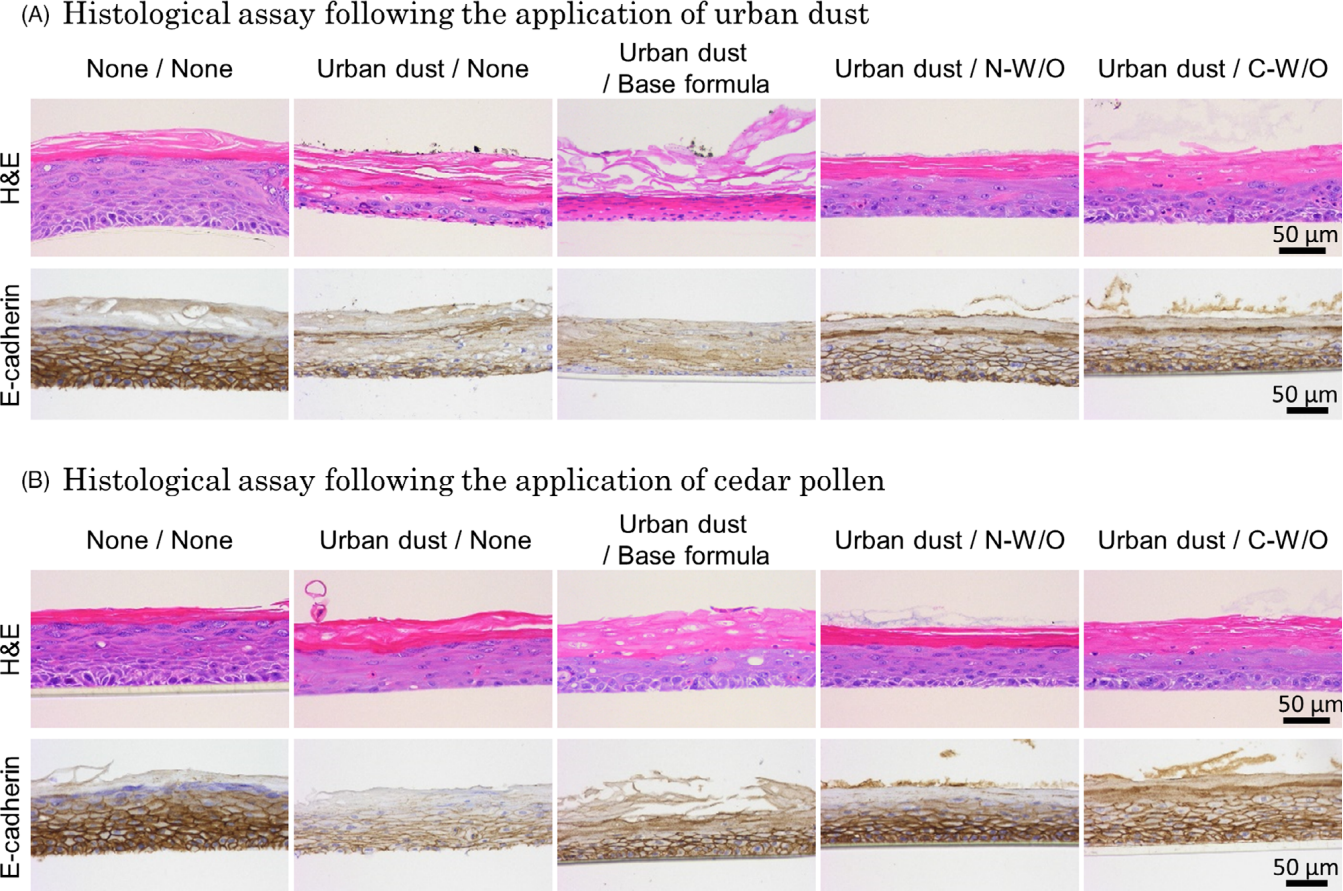
Histological assay by haematoxylin and eosin (H&E) staining and immunohistochemical staining of E‐cadherin at 24‐h exposure to urban dust (A) or cedar pollen (B). A thickened stratum corneum, thinned vital layer and cleaved E‐cadherin were observed with urban dust and cedar pollen exposure. N‐W/O prevented the degradation of epidermal integrity and intercellular adhesion.

Regarding the results of histological observations of RHE, a thickened stratum corneum and thinned vital layer were observed in the 24‐h PM‐exposed groups by H&E staining, and cleaved E‐cadherin in the epidermis was noted in these groups in the immunohistochemical analysis with the E‐cadherin antibody. These epidermal disruptive effects by PM exposure were more prominent in the urban dust‐treated group than in the cedar pollen‐treated group (Fig. 3a, b). N‐W/O and C‐W/O prevented the degradation of epidermal integrity more than the base formula in RHE exposed to PM (Fig. 3a‐e).

## Discussion

Particulate matter increased the mRNA expression levels of inflammation‐related genes and disrupted epidermal integrity in the RHE model. Gene expression and histological observations demonstrated that urban dust is a stronger inflammatory inducer than cedar pollen. Urban dust exerts markedly strong effects on skin because it contains a wide range of toxic contaminants, such as heavy metals, polycyclic aromatic hydrocarbons (PAHs), including benzo[a]pyrene (BaP), and endocrine‐disrupting chemicals. Furthermore, urban dust may penetrate skin more easily than cedar pollen because the diameters of 99% of urban dust were less than 10 µm and 40% were 2 µm 16, which are markedly smaller than that of cedar pollen (30 µm) 17.

Urban dust and cedar pollen significantly activated oxidative stress‐related pathways in the ranking based on the pathway enrichment analysis by MetaCore®. Approximately 50 % of up‐ and down‐regulated DEGs in urban dust and cedar pollen, such as *MMP1* and *CXCL8*, were the same. According to previous findings, PM induces oxidative stress through the formation of ROS 18, 19, 20, 21. PAHs localize in mitochondria in which they induce major structural damage 18, and they produce superoxide (O_2_^•−^) and the hydroxyl radical (•OH) by the redox cycling of NADPH‐cytochrome P450 reductase 19, 20. In addition, pollen grain extracts exhibit NADPH oxidase activity and NADPH oxidases increase O_2_^•−^ levels 21. These previous reports suggest that the similar effects on the gene expression and histological damage by urban dust and cedar pollen in this study were induced by ROS.

On the other hand, approximately 50% of up‐ and down‐regulated DEGs in urban dust and cedar pollen were different. Cytochrome P4501A1 (*CYP1A1*) and *CYP1B1* mRNA were more strongly induced in the urban dust‐treated group than in the cedar pollen‐treated group. BaP, which is composed of urban dust, is a ligand of the aryl hydrocarbon receptor (AhR), and AhR signalling has been shown to induce CYP1A1 and CYP1B1, which produce ROS 22, in skin and keratinocytes 23, 24, 25. Furthermore, *SLC7A11*, *SRXN1* and *PIR*, which were induced by urban dust 26, were detected as specific DEGs in urban dust samples. Cedar pollen exhibits serine protease activity 27, and Cry j1, a peptide allergen of cedar pollen, activates protease‐activated receptor 2 (PAR2) 28. *KRAS*, a gene that is up‐regulated by a PAR2 agonist, was only listed in the DEGs of the cedar pollen‐treated group 29. These findings suggested that ROS production is a common effect of urban dust and cedar pollen, and AhR and PAR2 signalling were specifically activated by urban dust and cedar pollen, respectively.

Matrix metalloproteinases and IL‐8 were chosen representative targets as proinflammatory factors that cause skin ageing because these targets are commonly induced by AhR and PAR2 agonists 11, 30, 31. In addition, MMP1 was selected as a typical matrix metalloproteinase related to skin ageing because it is elevated in the aged human skin and is responsible for the initiation of collagen fragmentation 32. The significant up‐regulation of these proinflammatory factors was significantly induced by PM exposure and prevented by N‐W/O treatment. Since these targets are associated with the early stages of skin inflammation and inflammatory factors were activated by the skin penetration of PM, other inflammatory factors induced by PM may also be prevented by N‐W/O treatment.

However, E‐cadherin mRNA was unchanged by PM exposure in this study, and the destruction of the epidermis was clearly observed by H&E staining. We demonstrated the immunohistochemical staining for E‐cadherin because it was known to be cleaved by MMP3 33 strongly induced by PM exposure. As a result, cleaved E‐cadherin was observed in PM‐treated groups. It was suggested that MMP3 induced by PM exposure in the epidermis cleaves E‐cadherin and causes skin destruction.

We previously reported that C‐W/O decreased the skin severity score, the frequency of scratching behaviour, and the grade of TEWL more effectively than petrolatum, which was used as a control, after 8 weeks of treatment for atopic dermatitis‐like skin symptoms in NC/Nga mice 13. The efficacy by which the induction of skin inflammation was prevented by N‐W/O tended to be higher than that by C‐W/O based on MMP1 and IL‐8 secretion and epidermal degradation.

In conclusion, N‐W/O made using natural surfactant is useful at protecting skin from PM, such as urban aerosols and cedar pollen.

## Conflict of Interest

None.
